# Galangin promotes cell apoptosis through suppression of *H19* expression in hepatocellular carcinoma cells

**DOI:** 10.1002/cam4.3195

**Published:** 2020-06-02

**Authors:** Xiaowei Zhong, Siyi Huang, Dianfeng Liu, Ziping Jiang, Qinglong Jin, Chengshun Li, Liu Da, Qunyan Yao, Dongxu Wang

**Affiliations:** ^1^ Laboratory Animal Center College of Animal Science Jilin University Changchun China; ^2^ Department of Hand Surgery The First Hospital of Jilin University Changchun China; ^3^ Department of Hepatology The First Hospital of Jilin University Changchun China; ^4^ Department of Pharmacy Changchun University of Chinese Medicine Changchun China; ^5^ Department of Gastroenterology and Hepatology Zhongshan Hospital Fudan University Shanghai China

**Keywords:** cell apoptosis, galangin, gene expression, H19, RNA‐seq

## Abstract

**Background:**

Galangin has been extensively studied as the antitumor agent in various cancers. However, the effect of galangin in hepatocellular carcinoma (HCC) remains elusive.

**Methods:**

Using RNA sequencing, the differential expression of lncRNA in human HCC cell line with highly metastatic potential (MHCC97H) cells treated with galangin was investigated. Furthermore, *H19* expression pattern was also determined in MHCC97H cells following treatment with galangin. In addition, knockdown and overexpression of *H19* was performed to analyze the effect of the expression pattern of *H19* on cell apoptosis, cell cycle, migration, and invasion in HCC cells. Moreover, the in vivo effect of galangin on tumor development was also determined in nude mice. In order to analyze loss expression of *H19* in vivo, clustered regularly interspaced short palindromic repeats/Cas9 (CRISPR/Cas9) was used.

**Results:**

Total of 50 lncRNAs were significantly differentially expressed in MHCC97H cells treated with galangin. Besides, the expression of *H19* was markedly reduced following treatment with galangin in MHCC97H cells. Compared to the Control group, the galangin‐treated group inhibited cell migration and invasion. Knockdown of *H19* expression showed increased cell apoptosis and decreased invasion. In addition, RNA‐seq data also identified 161 mRNA which was significantly differentially expressed following treatment with galangin. To further determine the underlying mechanism, p53 protein was analyzed. Notably, the results indicated that knockdown of *H19* and miR675 induced the expression of p53, eventually promoting cell apoptosis in MHCC97H cells. These results indicated that galangin promoted cell apoptosis through reduced the expression of *H19* and miR675 in MHCC97H cells. The in vivo result showed that compared to the Con, tumor growth was remarkably suppressed with loss expression of *H19*.

**Conclusion:**

Our data suggested that galangin has a crucial role in hepatocarcinogenesis through regulating the expression pattern of *H19*.

## Graphical abstract

Amount 50 lncRNAs were differentially expressed in galangin‐treated by RNA‐Seq. H19 induced cell apoptosis through p53 protein after galangin treatment. Target knock out H19 expression using CRISPR/Cas9.

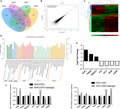

## 1 INTRODUCTION

HCC is the most cause of cancer deaths which was malignancy in liver. Long non‐coding RNAs (lncRNAs) have been identified as an effective modulator of carcinogenesis; besides, abnormal expression of lncRNAs has been related to the initiation, progression, and metastasis in HCC.^1^, ^2^ Indeed, lncRNA *H19*, a paternally imprinted gene, is recognized to have a key role in the carcinogenic process.^3^ In recent years, altered expression of *H19* has been demonstrated in various cancers including bladder cancer^4^ and nasopharyngeal carcinoma.^5^ miR675, microRNA embedded in the first exon 1 of *H19*, has shown to exert an oncogenic role in liver cancers.^6^, ^7^ Moreover, increasing evidence indicated that *H19* regulates the level of miR675; thus, *H19* can regulate a number of biological processes through miR675. Besides, studies have also suggested that the H19/miR675 axis may contribute to carcinogenesis through the oncogenic function of miR675.^8^, ^9^ However, aberrant expression of *H19* and miR675 can influence tumor cell behavior in HCC to remain elusive.

Galangin, a natural dietary flavonoid, is derived primarily from honey and root of *Alpinia officinarum* Hance (Zingiberaceae), which exhibits antimicrobial, antiperoxidative, anti‐inflammatory, and antitumor properties and is extensively used as a traditional medicine in China.^10^ Recently, galangin has been shown to have role in treating various cancer including HCC.^11^ Accumulating evidence suggested that galangin exerts antitumor effects through induction of cell apoptosis, inhibition of cell migration in kidney tumor.^12^ Moreover, galangin could inhibit the growth of human breast cancer cells MCF7 and induce cell apoptosis.^13^ A recent study also indicated that the anticancer activity of galangin regulated p53 expression in nasopharyngeal carcinoma (NPC) cells.^14^ Moreover, galangin could induce cell apoptosis via Caspase‐3 in retinoblastoma.^15^ These studies suggested that galangin has a crucial role in cell apoptosis.

Indeed, the major factor of liver cancer was metastasis. MHCC97H and HCC‐LM3 were both from HCC cell line with high metastatic potential (MHCC97).^16^ Our study focussed on migration and invasion of HCC cells. Moreover, MHCC97H and HCC‐LM3 were suitable for the analysis of the expression of genes and proteins. Thus, MHCC97H and HCC‐LM3 were selected. As herbal medicines, galangin (3,5,7‐trihydroxyflavone) was a potential drug for the treatment of HCC.^17^ There is evidence that galangin has benefits to reduce the risk of cancer.^18^ Previous report indicated that abnormal epigenetic modification and the expression of cancer‐related genes might contribute to HCC progression.^19^ For the treatment of HCC, screening of miRNA or lncRNA biomarkers is gradually becoming the hottest issues. In the present study, RNA sequencing was performed to analyze the differential expression of lncRNA. Furthermore, the expression of *H19* was determined in MHCC97H cells following treatment with galangin. The effect of knockdown and overexpression of *H19* on cell apoptosis, growth, cycle, migration, and invasion was also evaluated. Considering of CRISPR/Cas9 system is highly efficient for gene editing ^20^; thus, the effect of *H19* knock out (KO) on tumor development was also evaluated in vivo in nude mice. Our findings suggested that galangin has a significant role in hepatocarcinogenesis through regulating the expression of *H19*.

## 2 MATERIALS AND METHODS

### 2.1 Cell culture and drug treatment

Human HCC cell lines (MHCC97H, MHCC97L, and HCC‐LM3) were obtained from Liver Cancer Institute (Zhongshan Hospital, Fudan University).^21^ The cells were incubated in Dulbecco's modified Eagle's medium (DMEM; Gibco)—high glucose supplemented with 10% fetal bovine serum (FBS; Gibco) in a humidified atmosphere of 5% CO_2_ at 37°C. Prior to treatment, the cells were grown to 80%‐90% confluence. Then, MHCC97H and HCC‐LM3 cells (2 × 10^5^ cells/mL) were treated with galangin (50 μmol/L, Sigma, Purity ≥ 95%) for 48 hours.

### 2.2 RNA isolation and RNA‐seq analysis

MHCC97H cells were grown to 80%‐90% confluence; subsequently treated with galangin (50 μmol/L) for 48 hours. Total RNA of MHCC97H cells was extracted with TRIzol Reagent (Invitrogen). rRNA removal and subsequent purification were performed and with RiboZero Magnetic Gold Kit according to the instructions. RNA‐seq was carried out at the Sequencing and Non‐Coding RNA Program at the Sangon Biotech (Shanghai) on the HiSeq2500 (Illumina). Using HISAT2, RSeQC, BEDTools, and Qualimap, the reads were aligned and calculated the RPKM (reads per kilobase per million) values. The data submitted to Gene Expression Omnibus (GEO) dataset (GSE142680).

### 2.3 Knockdown and overexpression of *H19* and miR675

Synthetic RNA oligonucleotides targeting *H19* was obtained from RiboBio (Guangzhou). The siRNA target sequence was GCGGGTCTGTTTCTTTACT. pcDNA3.1‐H19 was procured from GenePharma (Shanghai, China). miR675‐3p mimics and inhibitor were obtained from RiboBio (Guangzhou).

The CRISPR/Cas9 plasmids were obtained from Addgene (px458). Protocols for sgRNA design and the procedures required for the in vitro transcription have been described previously.^20^ The sgRNA‐oligo sequences are listed in Table S1.

MHCC97H cells were transfected with si‐H19, pcDNA3.1‐H19, miR675‐3p‐mimics, miR675‐3p‐inhibitor, H19‐KO for 48 hours, respectively. Control cells were transfected with nonspecific or scrambled siRNA.

### 2.4 Gene expression analysis

Total RNA was isolated from MHCC97H, MHCC97L, HCC‐LM3 cells, and tumor samples using the TRNzol reagent (TIANGEN) and cDNA was synthesized using the FastKing RT Kit (TIANGEN) according to the instructions. Quantitative real‐time PCR (qPCR) was performed to measure gene expression with the SuperReal PreMix Plus (TIANGEN) on the BIO‐RAD iQ5 Multicolor Real‐Time PCR Detection System. The qPCR cycle profile was performed at 95°C for 15 minutes, followed by 40 cycles of denaturation at 95°C for 10 seconds, annealing at 60°C for 20 seconds. The *GAPDH* was used as an internal reference and the relative gene expression as fold change was calculated using the 2^−ΔΔCT^ method. The primer sequences are listed in Table S2.

### 2.5 Methylation pattern of* H19* DMR

The bisulfite sequencing PCR amplification was performed as described previously.^22^ Briefly, the genomic DNA of MHCC97H, MHCC97L, and HCC‐LM3 cells was isolated using the TIANamp Genomic DNA Kit (TIANGEN) and subjected to the CpGenome™ Turbo Bisulfite Modification Kit (Millipore) according to the instructions. Nested PCR was performed for the amplification of the *H19* differentially methylated regions (DMRs). The primer sequences are listed in Table S3.

### 2.6 Gene sequence analysis

The sequences of miR675 and *H19* exon1 are listed in Table S4 which obtained from http://asia.ensembl.org/index.html. The methylation sequence was analyzed using BiQ Analyzer software (http://biq‐analyzer.bioinf.mpi‐inf.mpg.de/tools/MethylationDiagrams/index.php).

### 2.7 Cell migration and invasion

The migration of the cells was assessed using a wound‐healing assay. Briefly, at 48‐h post‐transfection, 5 × 10^5^ cells were cultured. A scraped line was established with a 10 μL pipette tip and the remaining cells were cultured in serum‐free medium. After 12, 24, 48, and 72 hours at 37°C, cellular migration toward the scratched area was photographed using an inverted microscope.

Invasion assays were performed with Matrigel (BD Biosciences, USA). Briefly, cell transfectants were serum starved for 24 hours in DMEM containing 0.1% FBS. Subsequently, 3 × 10^4^ cells were added to the upper chamber of each well coated with 20 μL Matrigel, 0.5 mL of 10% FBS‐containing medium was added to the lower chamber. After incubation for 24 hours, cells that invaded to the lower membrane of the chamber were fixed with 4% paraformaldehyde, stained with 0.2% crystal violet dye (Solarbio). Then, the cells counted in five randomly selected fields (at × 200 magnification) under an inverted microscope. The average cell number per view was calculated. All experiments were performed in triplicate.

### 2.8 Cell counting kit‐8 assay

Cell viability was assessed with Cell Counting Kit‐8 (CCK‐8) assay kit (Dojindo, Kumamoto, Japan) as described previously.^23^ Briefly, cells were seeded at a density of 4 × 10^3^ cells/well. Following different treatments, 10 μL of CCK‐8 solution was added to each well. The cells were incubated for 30 minutes. The cell viability was revealed by the absorbance (OD), which was measured at 450 nm using a microplate reader (Infinite M200, TECAN).

### 2.9 Cell cycle and apoptosis analysis

To analyze the cell cycle, PI staining was performed. In brief, MHCC97H cells (1 × 10^6^ cells/mL) were treated with galangin, si‐H19, or pcDNA3.1‐H19 for 48 hours. The cells were washed using PBS and then fixed with 70% ethanol for 24 hours. These cells were incubated with PI and RNase A for 30 minutes and the fluorescence of the cells was quantified by flow cytometry (BD Biosciences) using a PI signal detector (BD Accuri^TM^ C6).

The cell apoptosis analysis was performed as previously described.^24^ Briefly, MHCC97H cells were treated with galangin, si‐H19, or pcDNA3.1‐H19 for 48 hours and then washing twice using PBS. The harvested cells (1 × 10^6^ cells/mL) were incubated with a mixture of Annexin V‐FITC/PI for 30 minutes following the manufacturer's protocol. A FITC signal detector and a PI signal detector (BD Accuri C6) were used to quantify the fluorescence of the cells by flow cytometry (BD Biosciences).

### 2.10 Western blot analysis

The proteins were extracted from cell lines (1 × 10^6^ cells) in ice‐cold protein extraction buffer (Novagen, Madison, WI, USA) with 2 × SDS lysis buffer supplemented with protease inhibitors cocktail. BCA protein assay kit (TIANGEN) was used to quantify the concentrations of the protein. For western blot assay, proteins were separated by 10% sodium dodecyl sulfate‐polyacrylamide gel electrophoresis (SDS‐PAGE) followed by transfer onto a polyvinylidene difluoride (PVDF) membrane. Subsequently, membranes were blocked with 5% non‐fat milk in Tris‐buffered saline with Tween‐20 (TBS‐T; 0.1% Tween‐20 in TBS) and probed with primary antibodies against anti‐p53 (Bioworld, BS9809M) and anti‐GAPDH (Affinity, AF7021), respectively, each at a dilution of 1:2000 in 5% blocking buffer overnight at 4°C. Subsequently, the membranes were washed twice using TBS‐T and incubated with horseradish peroxidase (HRP)‐conjugated secondary antibodies (anti‐mouse or anti‐rabbit, Boster) for 1 hour at room temperature. The target bands of proteins were visualized using ECL Super Signal software (Pierce).

### 2.11 Nude mice xenograft assay

The animals were cared for in accordance with the Guide for the care and use of laboratory animals in China. All experimental procedures were approved by the Animal Care and Use Committee of Jilin University (Grant No. SY201907008). In all, 30 female nude mice (6‐week old) were procured from the Laboratory Animal Center, Jilin University. The mice were randomly divided into six groups (N = 5). Con, pcDNA3.1‐H19, and H19‐KO cells (3 × 10 ^5^ cells) were injected subcutaneously into the left flank areas of mice. The mice tumors were observed after 11 days. The mice were used for experiments when their tumor volumes were approximately 40‐60 mm^3^. The galangin group was administrated with galangin (Sigma) by daily gavage at 20 mg/kg for 14 days. The Con group was given equal quantities of saline. The length (*L*) and width (*W*) were recorded and the tumor volumes were calculated as (*L* × *W* ^2^/2). The mice were housed in laboratory cages under controlled laboratory conditions, at 24°C under a 12‐hour light/dark cycles. Animals were provided ad libitum access to standard rodent food and tap water. All the mice were healthy and had no infection during the experimental period. All surgical procedures were carried out under aseptic conditions.

### 2.12 Statistical analysis

All data are presented as mean ± SD using GraphPad Prism 5.0 (GraphPad Software, Inc). The Student *t* tests (Unpaired *t* test) were used to analyze the data. A *P*‐value of < .05 was considered statistically significant.

## 3 RESULTS

### 3.1 Analysis of lncRNAs expression profiles by high‐throughput RNA‐seq

**FIGURE 1:**
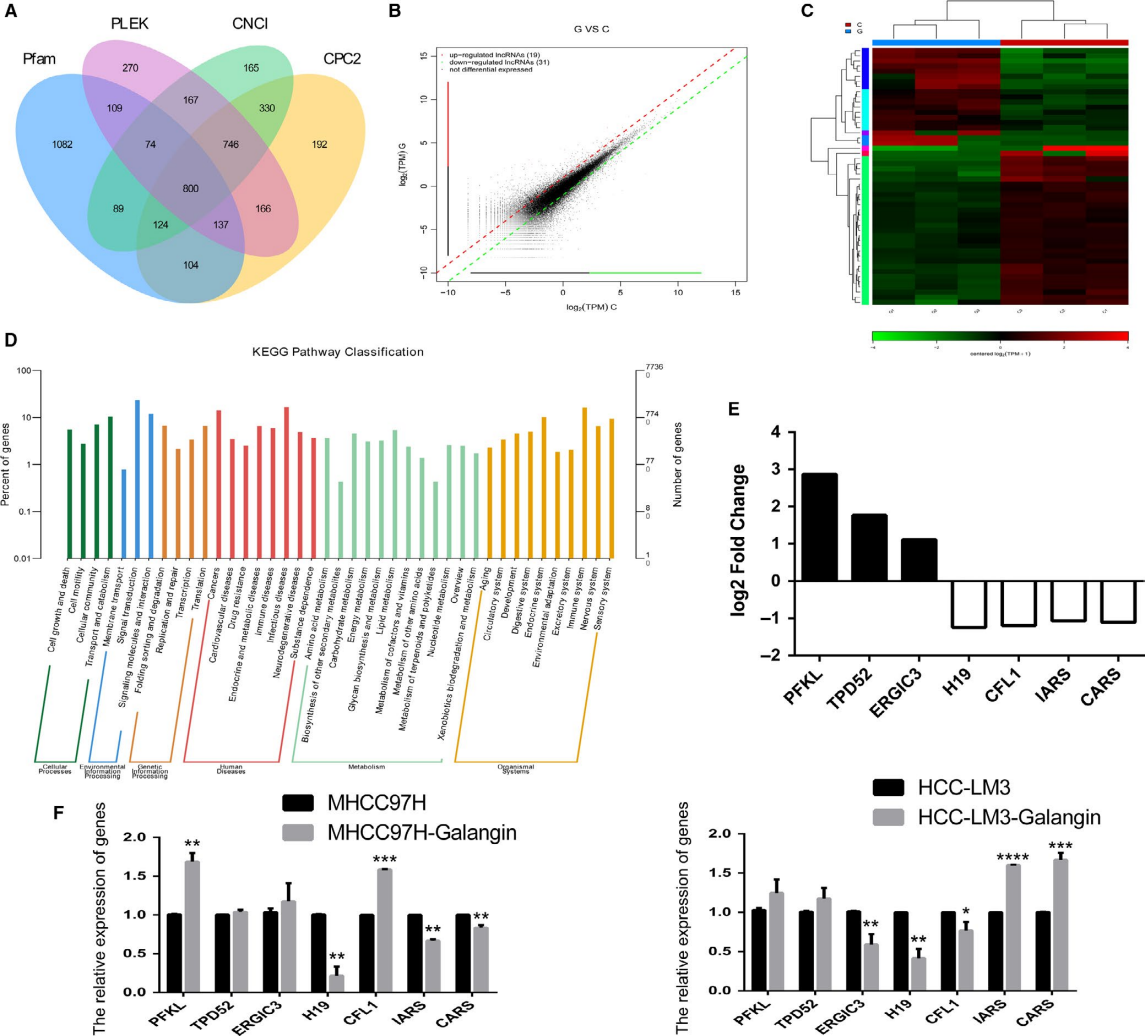
LncRNAs expression profile by RNA‐seq. Screen lncRNAs using the software of CPC2, CNCI, Pfam, and PLEK (A). Identification of different expressed lncRNAs (B). The heatmap was drawn to show the differentially expressed lncRNAs (C). KEGG pathway of the differentially expressed lncRNAs (D). The expression of log2 fold change in lncRNAs (E). Relative expression of *PFKL, TPD52, ERGIC3, CFL1, CARS, IARS*, and *H19* was analyzed by qPCR after galangin treatment in MHCC97H and HCC‐LM3 cells (F). C, indicated Control group. G, indicated galangin treatment group. The data are represented as the mean ± SD (n = 3). *(*P* < .05), **(*P* < .01), ***(*P* < .005), and ****(*P* < .001) indicate statistically significant differences

To analyze the lncRNA expression pattern after treatment with galangin in MHCC97H cells, RNA‐Seq was performed with Illumina Hiseq. A total of 800 lncRNAs were identified using the software programs *CPC2*, *CNCI*, *PFAM*, and *PLEK* (Figure 1A). Compared to the Control group, 50 lncRNAs were differentially expressed in galangin‐treated group (Figure 1B). The heatmap and Kyoto Encyclopedia of Genes and Genomes (KEGG) pathways revealed that the differentially expressed lncRNAs have role in cell growth and death (Figure 1C,D). Furthermore, we focused on the seven lncRNAs (*PFKL, TPD52, ERGIC3, CFL1, CARS, IARS*, and *H19*), which were related to cancer development (Figure 1E). To further detect the expression of these genes, qPCR was used in MHCC97H and HCC‐LM3 cells. The result showed only the expression of *H19* was reduced after galangin treatment in both MHCC97H and HCC‐LM3 cells (Figure 1F).

### 3.2 Galangin induces cell apoptosis and suppresses cell migration and invasion

**FIGURE 2:**
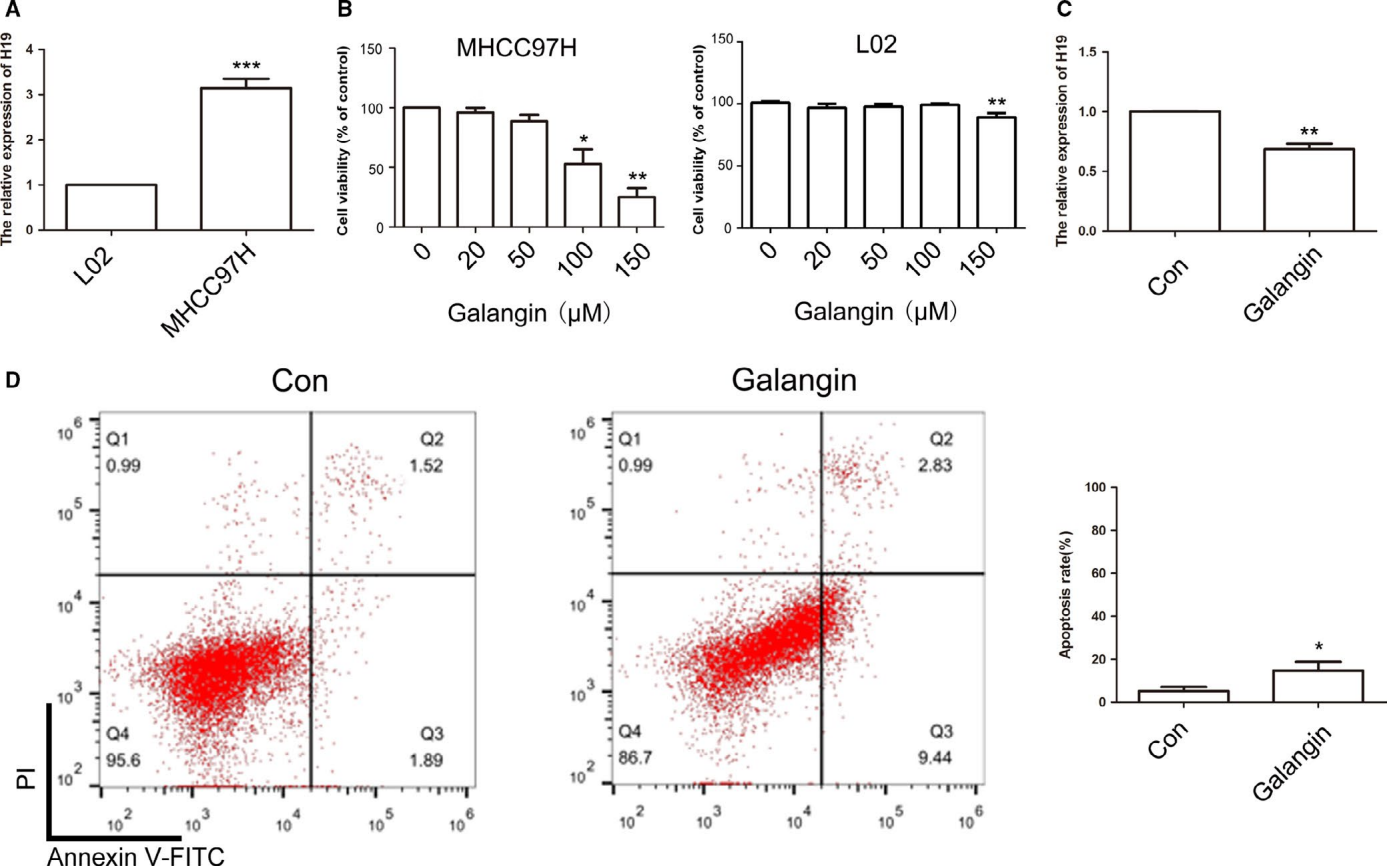
Analysis of *H19* expression and cell apoptosis after galangin treatment. Relative expression of *H19* between L02 and MHCC97H cells (A). The cell growth was analyzed by CCK8 assay (B). Relative expression of *H19* was analyzed by qPCR after galangin treatment in MHCC97H cells (C). The cell apoptosis was analyzed between Con and galangin group (D). Statistical analysis of the percentage of cell apoptosis (E). The data are represented as the mean ± SD (n = 3). *(*P* < .05), **(*P* < .01), and ***(*P* < .005) indicate statistically significant differences

**FIGURE 3:**
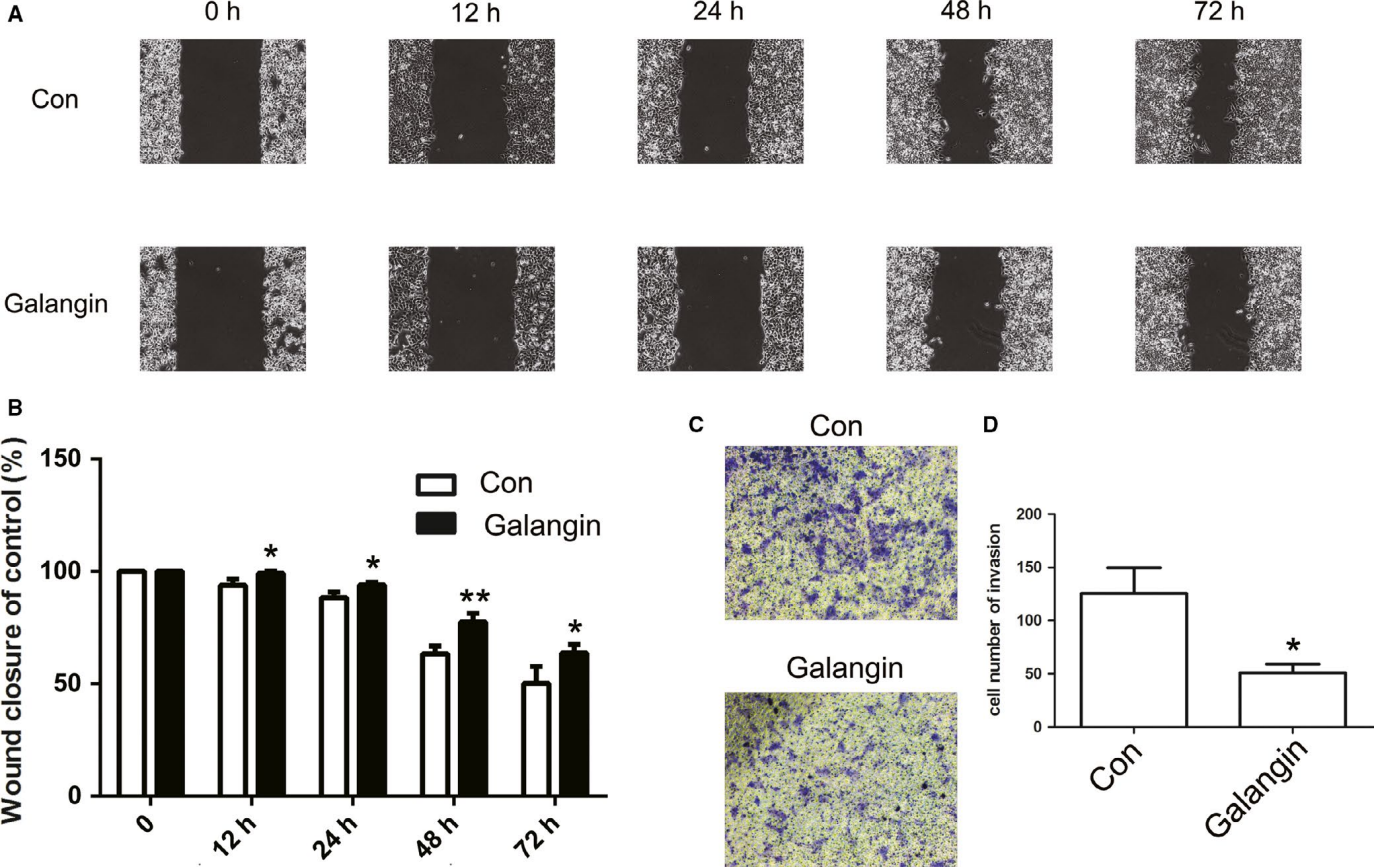
Analysis of cell migration and invasion after galangin treatment. The cell migration was analyzed between Con and galangin group (A). Statistical analysis of the percentage of cell migration (B). The cell invasion was analyzed (C). Statistical analysis of the percentage of cell invasion (D). The data are represented as the mean ± SD (n = 3). *(*P* < .05) and **(*P* < .01) indicate statistically significant differences

qPCR results suggested that *H19* was significantly overexpressed in MHCC97H as compared to L02 cells (Figure 2A). L02 cells derived from normal human liver tissue. To further confirm the expression pattern of *H19*, MHCC97L and HCC‐LM3 cells were also analyzed. qRT‐PCR and BSP results identified the aberrant expression of *H19* and hypo‐methylation pattern of *H19* DMR in MHCC97H, MHCC97L, and HCC‐LM3 cells (Figure S1). To determine if treatment with galangin affected the MHCC97H cell growth, CCK8 assay was carried out. As illustrated in Figure 2B, treatment with 100 and 150 μmol/L of galangin exhibited toxic effects on MHCC97H cells. Thus, 50 μmol/L of galangin was selected for qPCR analysis. The qPCR findings revealed reduced expression of *H19* after treatment with galangin (Figure 2C). Moreover, galangin could induce significant cell apoptosis in MHCC97H cells compared with the control cells (Figure 2D,E). Flow cytometry analysis showed reduced S phase cells in galangin‐treated cells (Figure S2). Furthermore, treatment with galangin inhibited the cell migration (Figure 3A,B) and invasion (Figure 3C,D) of MHCC97H cells, indicating that galangin can serve as a potential antitumor agent.

### 3.3 Effects of knockdown and overexpression of *H19* on cell apoptosis

**FIGURE 4:**
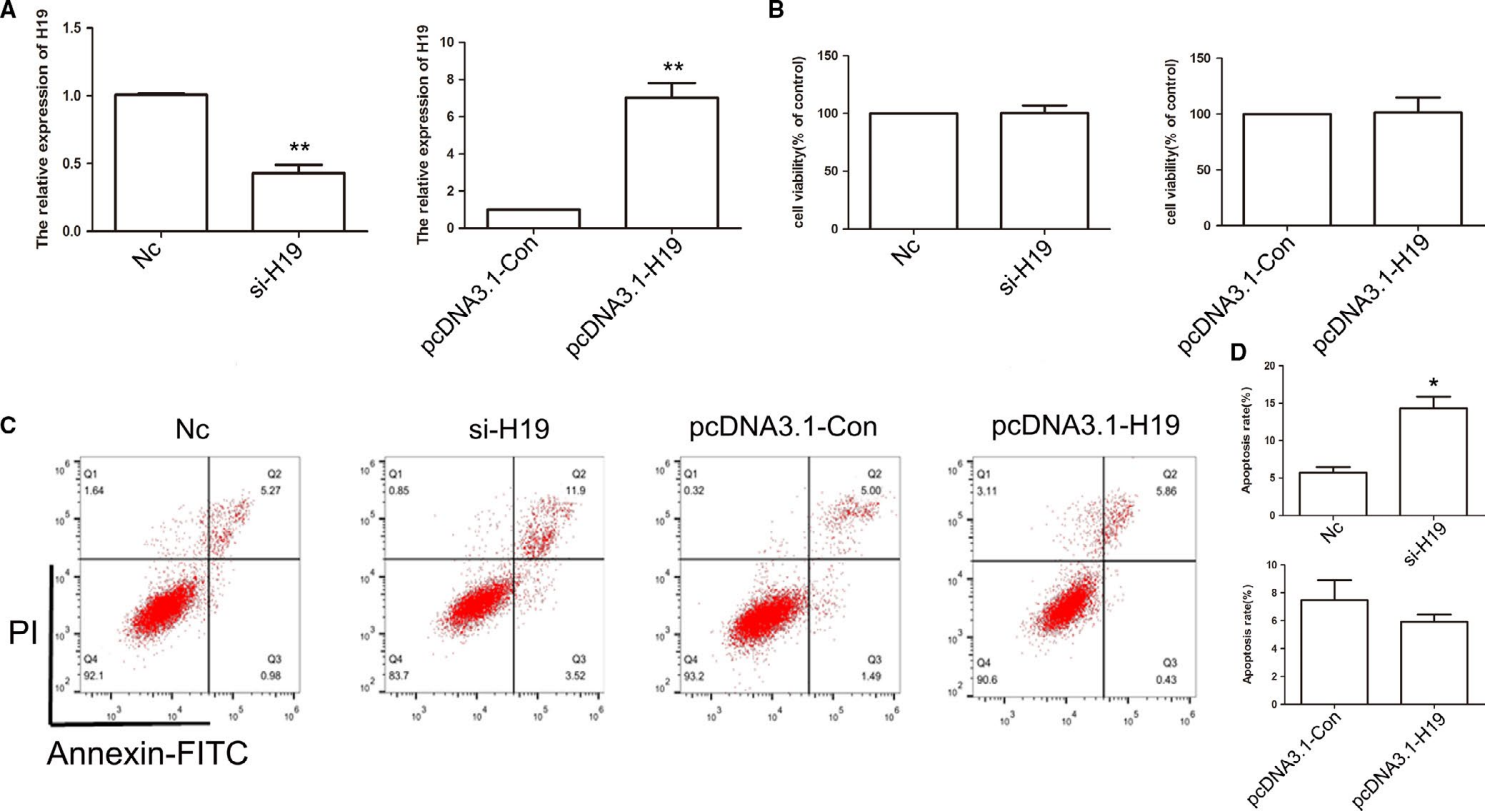
Analysis of *H19* expression pattern in MHCC97H cells. Relative expression of *H19* in Nc, si‐H19, pcDNA3.1‐Con, and pcDNA3.1‐H19 group using qPCR (A). The cell growth was analyzed by CCK8 assay (B). The cell apoptosis was analyzed after si‐H19 and pcDNA3.1‐H19 transfected (C). Statistical analysis of percentage of cell apoptosis (D). The data are represented as the mean ± SD (n = 3). *(*P* < .05) and **(*P* < .01) indicate statistically significant differences

**FIGURE 5:**
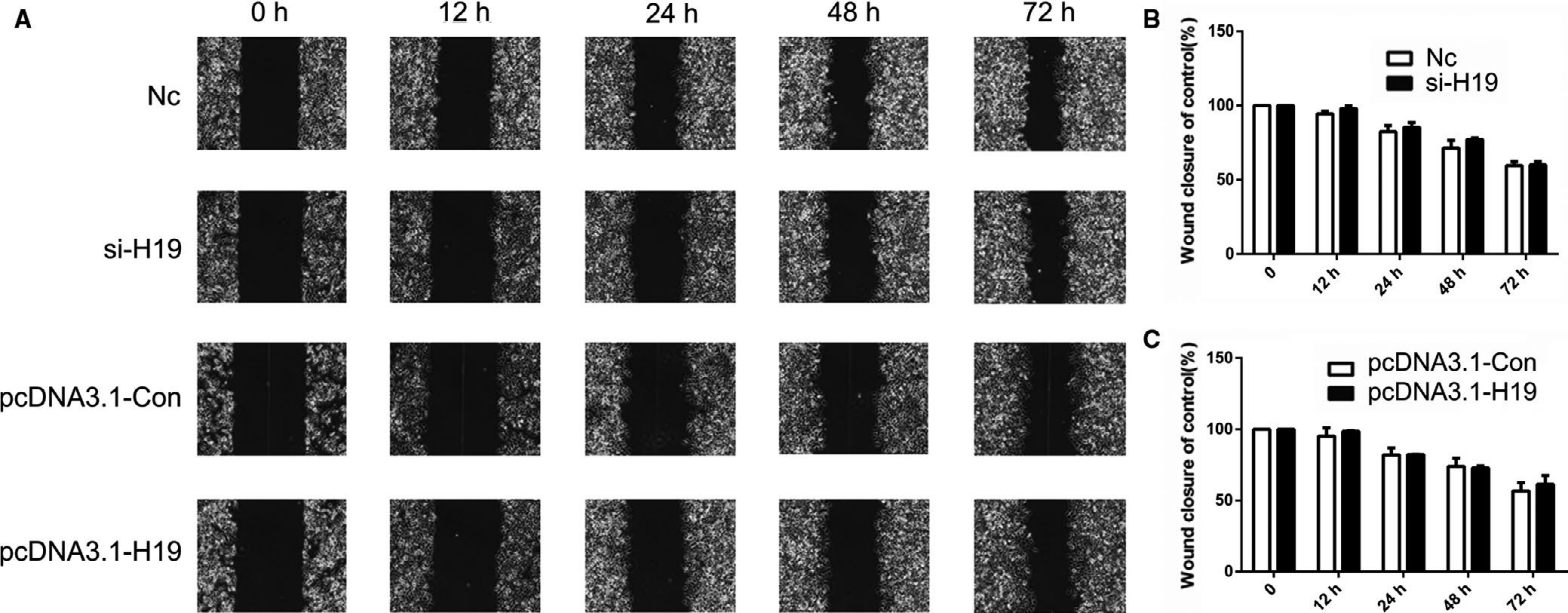
Analysis of cell migration after knockdown and overexpression of *H19*. The cell migration was analyzed in Nc, si‐H19, pcDNA3.1‐Con, and pcDNA3.1‐H19 group (A). Statistical analysis of the percentage of cell migration (B and C). The data are represented as the mean ± SD (n = 3)

**FIGURE 6:**
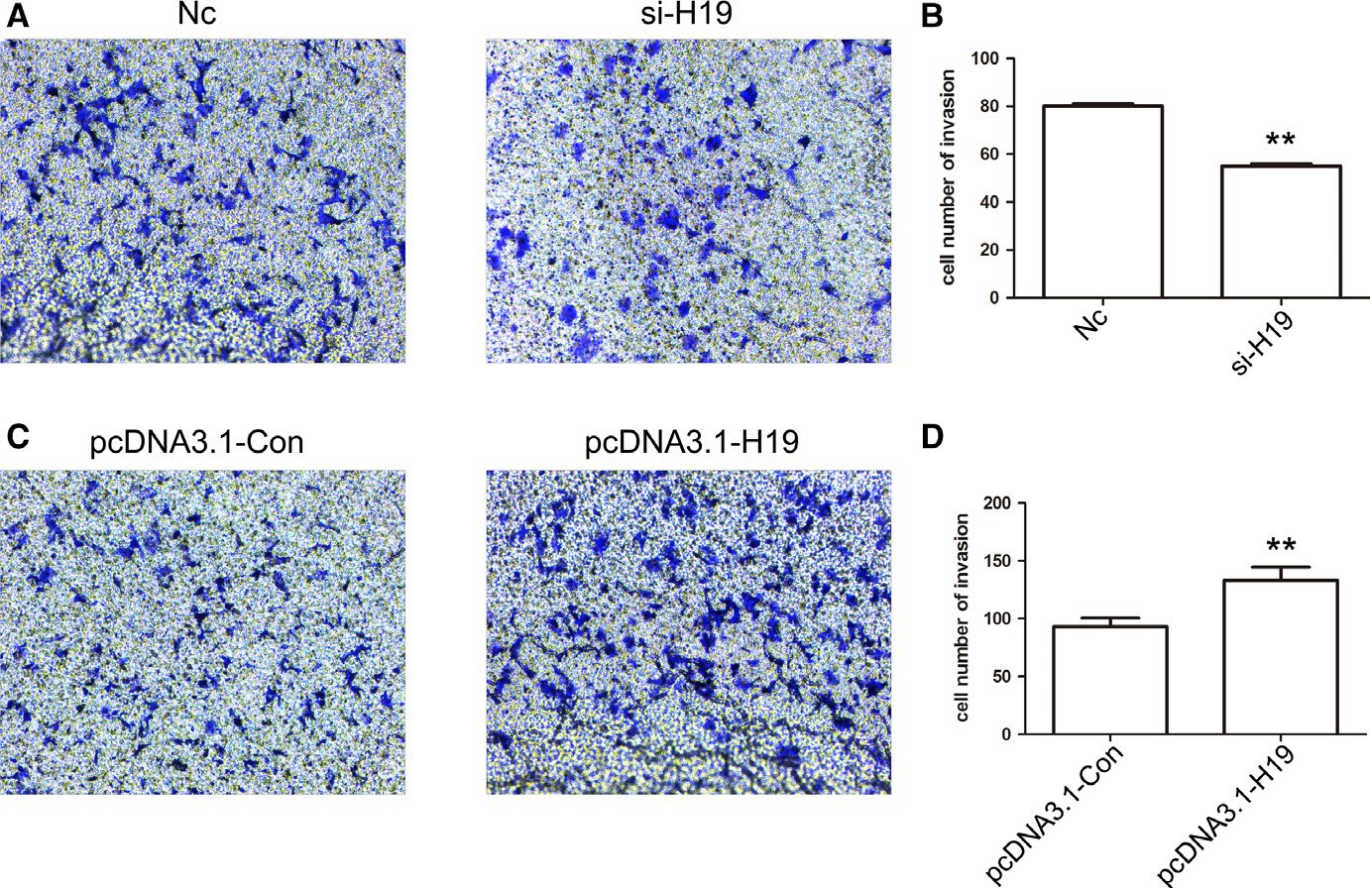
Analysis of cell invasion after knockdown and overexpression of *H19*. The cell invasion was analyzed in Nc, si‐H19, pcDNA3.1‐Con, and pcDNA3.1‐H19 group (A and C). Statistical analysis of the percentage of cell migration (B and D). The data are represented as the mean ± SD (n = 3). **(*P* < .01) indicate statistically significant differences

To further analyze the effect of the expression pattern of *H19* on cell apoptosis, HCC cells were transfected with si‐H19 or pcDNA3.1‐H19 expression vector. The qPCR results revealed reduced expression of *H19* in the siRNA‐treated group, while overexpression was observed in the pcDNA3.1‐H19 expression group (Figure 4A). CCK8 assay indicated that *H19* expression could not alter the cell growth (Figure 4B). However, cell apoptosis results indicated that reduced expression of *H19* induced marked cell death in MHCC97H cells (Figure 4C,D). Besides, *H19* expression did not alter the cell cycle (Figure S3). These data suggested that *H19* expression exhibits crucial roles in cell apoptosis. To further investigate the effect of *H19* expression pattern in MHCC97H cells, cell migration and invasion were analyzed. Cell migration result indicated that compared to the control group, knockdown or overexpression of *H19* did not show any alteration (Figure 5A‐C). However, the cell invasion result showed that reduced expression of *H19* could suppress the invasive potential of MHCC97H cells (Figure 6A‐D).

### 3.4 *H19*/miR675‐mediated cell apoptosis through p53 protein

**FIGURE 7:**
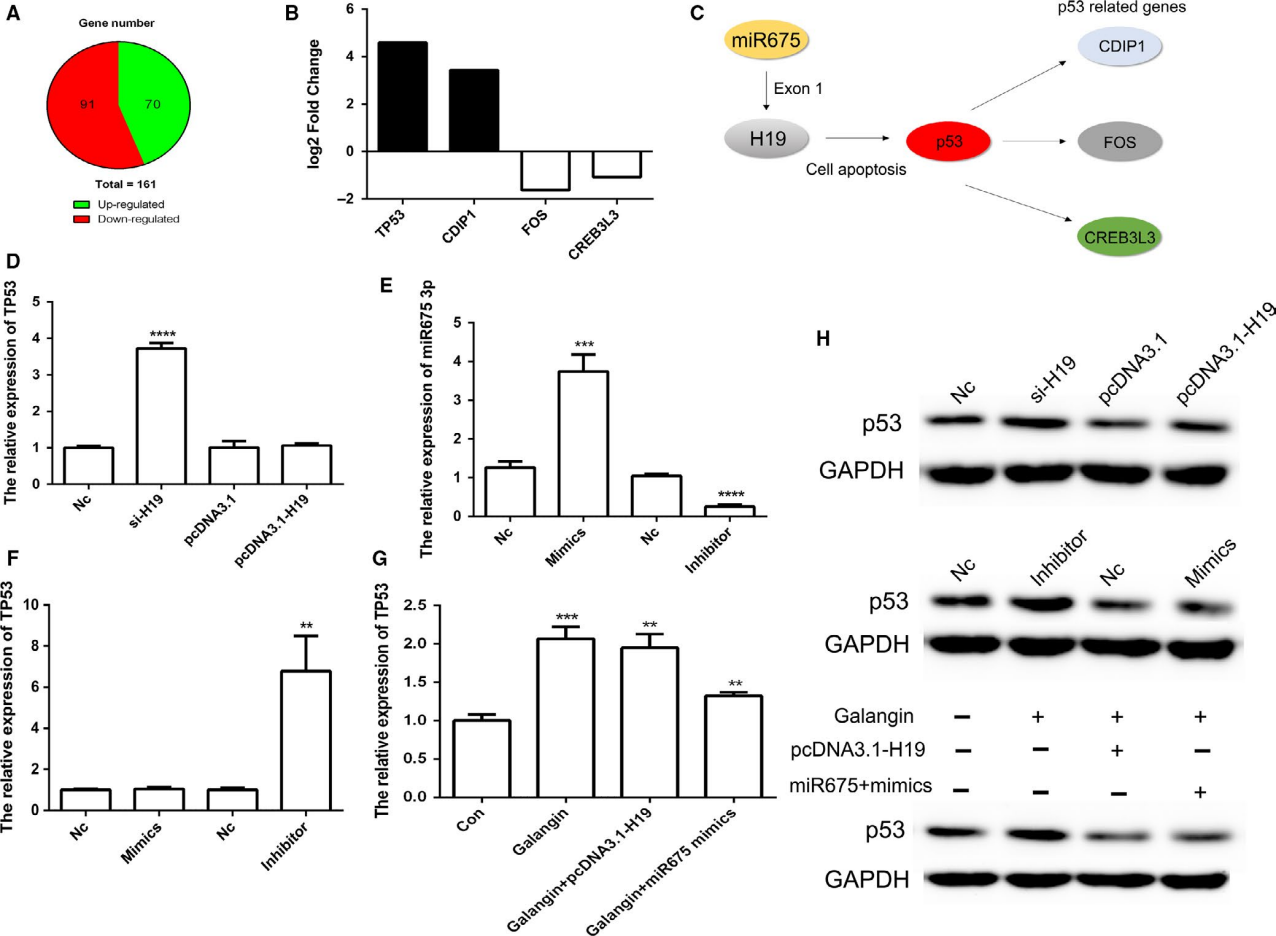
The expression pattern of *H19* and miR675‐3p in cell apoptosis. Screen of different expressed mRNA (A). The expression of log2 fold change in p53 and p53‐related genes (B). Schematic representations of *H19* and miR675 in p53 signaling pathway (C). The relative expression of *TP53* in Nc, si‐H19, pcDNA3.1‐Con, and pcDNA3.1‐H19 group (D). The relative expression of miR675‐3p (E). The relative expression of *TP53* in Nc, miR675‐3p mimics and inhibitor group (F). The relative expression of *TP53* after galangin treatment (G). Analysis of the expression of p53 protein using Western blot (H). The data are represented as the mean ± SD (n = 3). **(*P* < .01), ***(*P* < .005), and ****(*P* < .001) indicate statistically significant differences

RNA‐Seq data showed that compared to the control group, 161 mRNA were differentially expressed in galangin‐treated group (Figure 7A). Analysis of cell apoptosis signaling pathway revealed that mRNA of *TP53*‐ and p53*‐*related genes (*CDIP1, FOS,* and *CREB3L3*) were significantly differentially expressed following treatment with galangin (Figure 7B). Moreover, miR675‐3p, which locus in *H19* exon 1 as potential targeting of p53, was investigated (Figure 7C). qPCR result suggested that reduced expression of *H19* increased *TP53* expression (Figure 7D). Furthermore, increased expression of *TP53* was observed after transfection with miR675 inhibitor (Figure 7E,F). Moreover, the expression of *TP53* was increased by treatment with galangin after transfection with pcDNA3.1‐H19 or miR675 mimics (Figure 7G). Western blot analysis further confirmed our qPCR data (Figure 7H). Taken together, these results indicated that p53 protein was regulated by the *H19*/miR675 axis.

### 3.5 Galangin inhibited tumor growth in vivo

**FIGURE 8:**
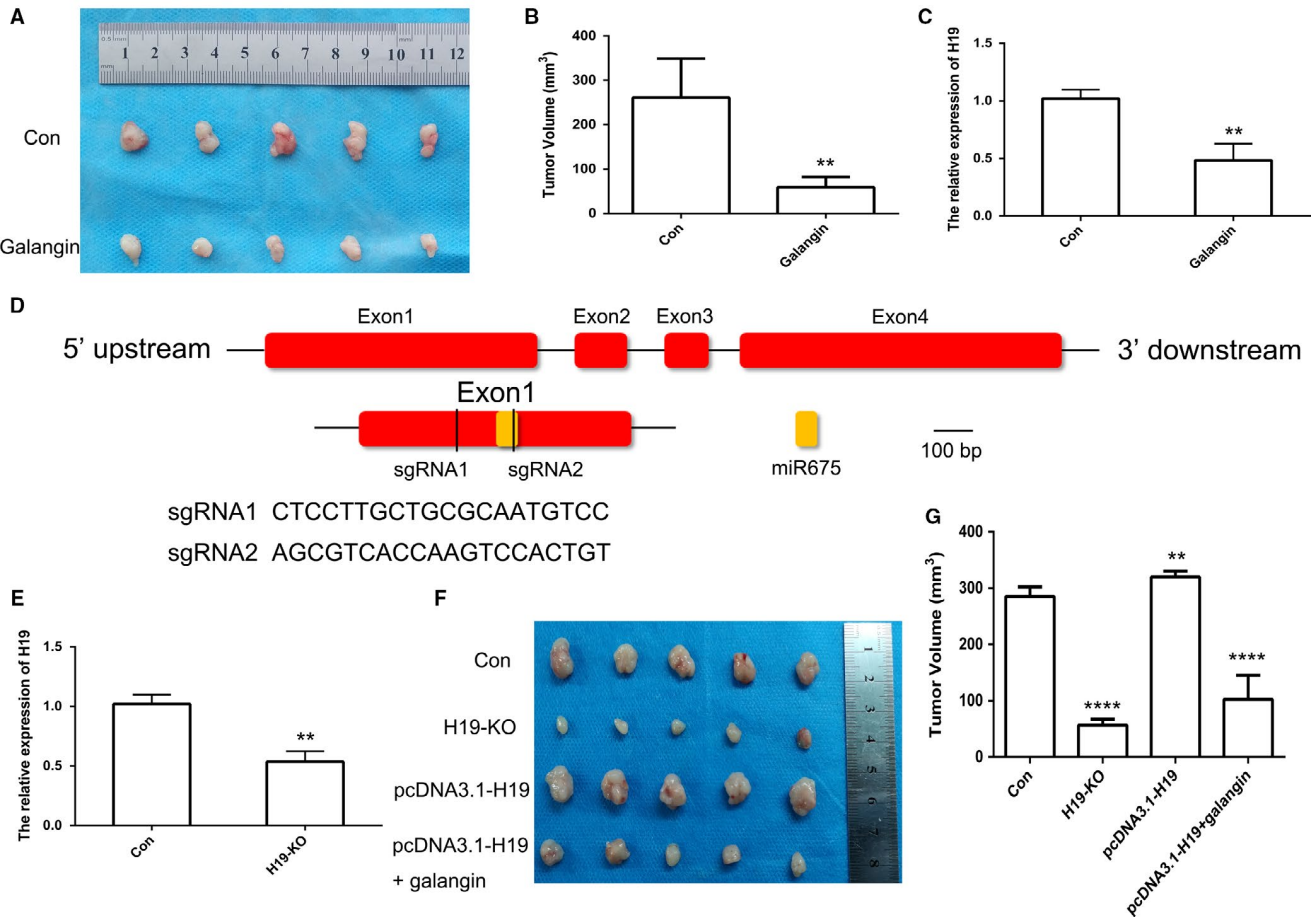
CRISPR/Cas9‐mediated gene targeting of *H19*. Morphological observation of mouse HCC tumor tissue (A). Analysis of tumor volume (B). The expression pattern of *H19* in tumor of mice after galangin treatment (C). Schematic diagram of sgRNA targeting the *H19* gene loci (D). The expression of *H19* using qPCR (E). The tumor morphology (F) and volume (G). Red indicated exon of *H19*. Yellow indicated miR675. **(*P* < .01) and ****(*P* < .001) indicate statistically significant differences

To confirm the galangin‐meditated *H19* expression in vivo, nude mice xenograft was used. After 11 days, the mice were treated with galangin (20 mg/kg) or an equal volume of saline for 14 days. The results indicated that galangin had a significant inhibitory effect on tumor growth (Figure 8A,B). qPCR results revealed that treatment with galangin could significantly inhibit the expression of *H19* in vivo (Figure 8C). In order to analyze loss expression of *H19* in MHCC97H cells, two sgRNAs targeting the exon1 of *H19* were designed (Figure 8D). qPCR result suggested that *H19* expression was reduced in *H19* KO cells (Figure 8E). To analyze the effect of the expression pattern of *H19* in vivo, the *H19* KO cells were injected into nude mice. The results demonstrated that inhibited tumor growth was observed after injected *H19* KO cells compared with control group. Moreover, galangin treatment could inhibit tumor growth in *H19* overexpression group (Figure 8F‐G). These findings indicated that *H19* which can be regulated by galangin might have an important role in the development of cancer.

## 4 DISCUSSION

In recent years, lncRNAs have received increased attention as a novel and crucial player in various cellular processes. Aberrant expression of lncRNAs was associated with cancer development. As potential biomarkers of cancer, lncRNA regulated tumor suppressors or oncogenes through binding to DNA, RNA, or proteins.^25^ Moreover, RNA‐Seq data have revealed subsets of lncRNA whose expression patterns were found to be substantially associated with malignancy in several types of cancer including HCC. In this context, previous studies have revealed that *TPTEP1*, a lncRNA, exhibits a crucial role in HCC by RNA‐seq analysis.^26^ Indeed, many lncRNAs, such as *LINCO1138* and *lncAKHE*, have been found to participate in the development of HCC.^27^, ^28^ In this study, using RNA‐seq analysis, we investigated the differential expression of lncRNAs following treatment with galangin in MHCC97H cells. The total of 50 lncRNAs were identified which were differentially significant expressed. Furthermore, we analyzed cancer‐related lncRNAs in the data and our analysis revealed that compared to L02 cells, *H19* expression was dramatically increased in MHCC97H and HCC‐LM3 cells. The *H19* expression was mediated by DNA methylation.^29^ Consistently, our data further indicated the aberrant methylation status of *H19* DMR in HCC cells.

Considering of HCC is a multistage process which involved with epigenetic modification and the expression of lncRNAs, novel therapeutic drug and biomarkers are still urgently needed. Numerous genes and proteins which control cell proliferation, invasion, and metastatic formation have role in HCC pathogenesis. Chemoprevention is to treat cancer with nontoxic natural or synthetic chemicals, such as galangin.^30^ Chemoprevention has ability to regulate numerous gene and protein expressions with molecular targets and antitumor effects. Galangin as natural bioflavonoid primarily extracted from Chinese medicinal herb has been reported to have a role in oxidative stress and inflammation through regulating the gene expression of cellular signaling pathways.^31^ Moreover, galangin has been recently proven to be an effective drug for the treatment of HCC which induced cell apoptosis.^11^ However, there is little evidence on galangin regulating the expression of *H19*. Our data indicated that the expression of *H19* was regulated by galangin indicating that galangin might be involved in cell apoptosis through regulating the expression of *H19* in MHCC97H cells. Previous reports also suggested that galangin could induce apoptosis via endoplasmic reticulum stress in PLC/PRF/5 cells.^32^ Our results confirmed that galangin significantly induced cell apoptosis in MHCC97H cells. Furthermore, the effect of treatment with galangin was analyzed in vivo and results indicated that tumor growth was markedly inhibited upon treatment with galangin. Previous reports also suggested that reduced *H19* expression was associated with tumor development.^33^ Our in vivo result confirmed that *H19* expression was noticeably suppressed after treatment with galangin. There is evidence that galangin could induce cell apoptosis through regulating the expression of p53 protein which is in accordance with our data in HCC cells.^14^ These results indicated that galangin has the ability to regulate *H19* expression which might induce cell apoptosis through p53 protein.

Furthermore, previous studies have also shown that *H19* expression has role in cell growth and invasion.^34^ Recent study suggested that knockdown of *H19* expression which regulated the CDC42/PAK1 pathway could inhibit cell growth, migration, invasion, and promote apoptosis through miR‐15b in HCC cells and tissues.^2^ Moreover, reduced expression of *H19* could induce cell apoptosis in HCC cells and other cancer cells.^1^, ^35^ Apparently, our result also suggested knockdown of *H19* expression induced apoptosis which further confirmed the previous findings. These results indicated that reduced expression of *H19* significantly promoted apoptosis in MHCC97H cells. Accumulating studies have also confirmed that migration and invasion of HCC were crucial in the prognosis of patients with HCC.^36^, ^37^ The previous report also revealed that lncRNAs, such as *HOXD‐AS1* and *EIF3J‐AS1*, play a role in migration and invasion of HCC.^38^, ^39^ Consistently, our result also revealed that *H19* expression was associated with invasion in MHCC97H cells.

RNA‐seq data revealed a total of 161 mRNA were differentially expressed after treatment with galangin. Of these, only *TP53*‐ and p53‐related genes (*CDIP1, FOS,* and *CREB3L3*) were associated with cell apoptosis.^40^ Increasing reports also suggested the expression of miR675 and *H19* had role in cell apoptosis through p53 protein in cancer cells.^41^, ^42^, ^43^ To confirm the putative function of *H19* and miR675 in cell apoptosis, overexpression and knockout of *H19* and miR675 were performed. Previous report suggested that CRISPR/Cas9 system was useful for gene editing in gastric cancer cells.^44^ Results of our study revealed that *H19* KO via CRISPR/Cas9 system could inhibit tumor growth. The result indicated that reduced expression of miR675 and *H19* promoted apoptosis through up‐regulating the protein expression of p53.

## 5 CONCLUSION

In this study, a total of 50 lncRNAs and 161 mRNA were identified to be differentially expressed in MHCC97H cells following treatment with galangin. The findings also demonstrated that *H19* expression is reduced by galangin in MHCC97H cells. Furthermore, knockdown of *H19* and miR675 induced the protein expression of p53, eventually promoting cell apoptosis. Collectively, our data suggested that galangin has role in hepatocarcinogenesis through regulating expression pattern of *H19*.

## CONFLICTS OF INTEREST

No potential conflict of interest was reported by the authors.

## AUTHORS’ CONTRIBUTION

Dongxu Wang designed the experiments and wrote the manuscript. Xiaowei Zhong, Siyi Huang, and Chengshun Li performed cell experiment and gene expression analysis. Qunyan Yao, Da Liu, and Dianfeng Liu contributed reagents and materials. Ziping Jiang carried out animal experiment. Qinglong Jin analyzed the data and prepared figures. All authors reviewed the manuscript.

## Supporting information

Fig S1

Fig S2

Fig S3

Table S1‐S4

## Data Availability

The data that support the findings of this study are available from the corresponding author upon reasonable request.
