# Supplementary figures and images for: Galangin promotes cell apoptosis through suppression of H19 expression in hepatocellular carcinoma cells
Source: Cancer Med. 2020 Jun 2;9(15):5546–57. doi: 10.1002/cam4.3195 (PMC7402821; doi:10.1002/cam4.3195)

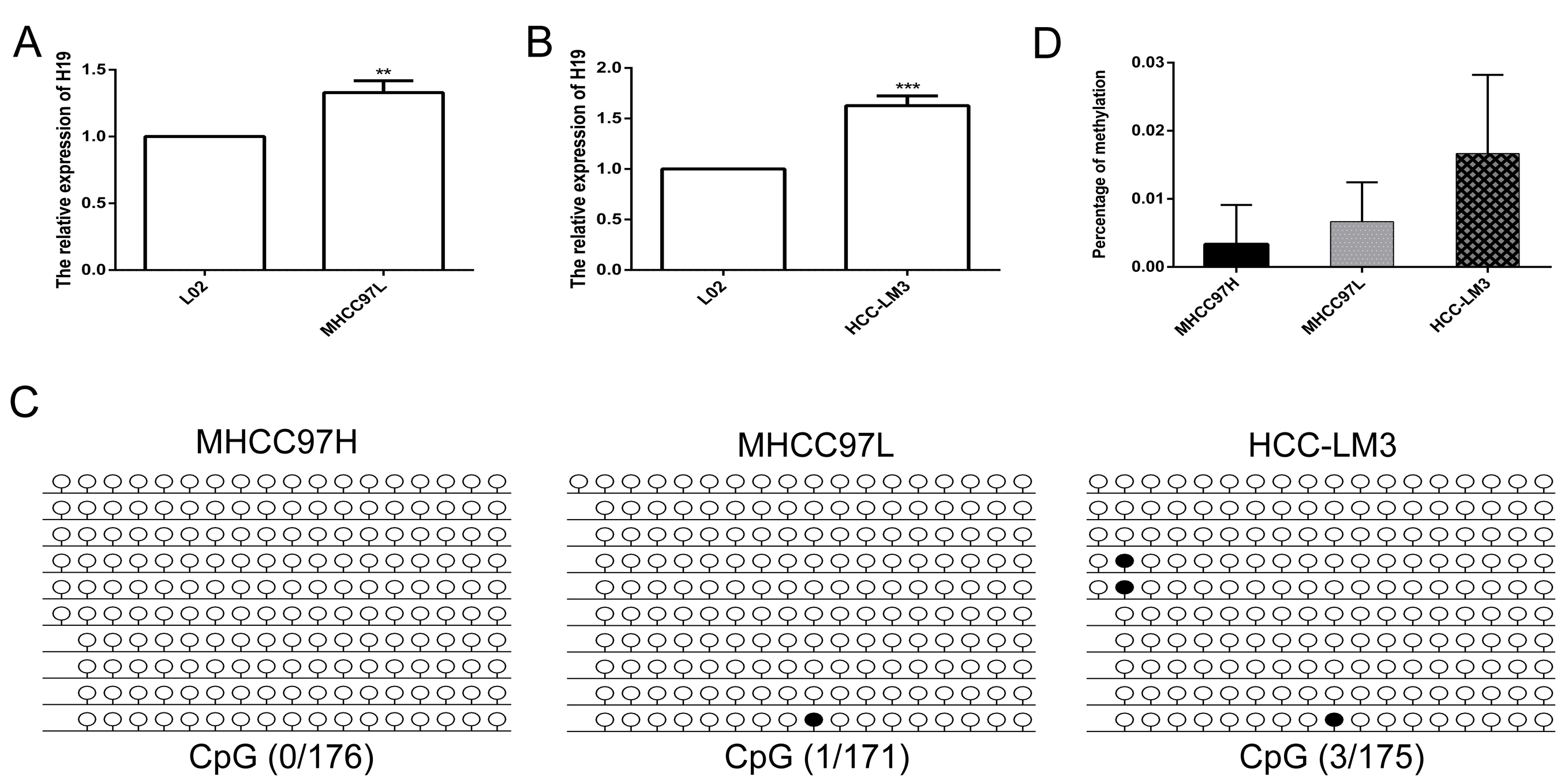

Supplement: Supplementary file 1 — Fig S1 [file CAM4-9-5546-s001.tif]

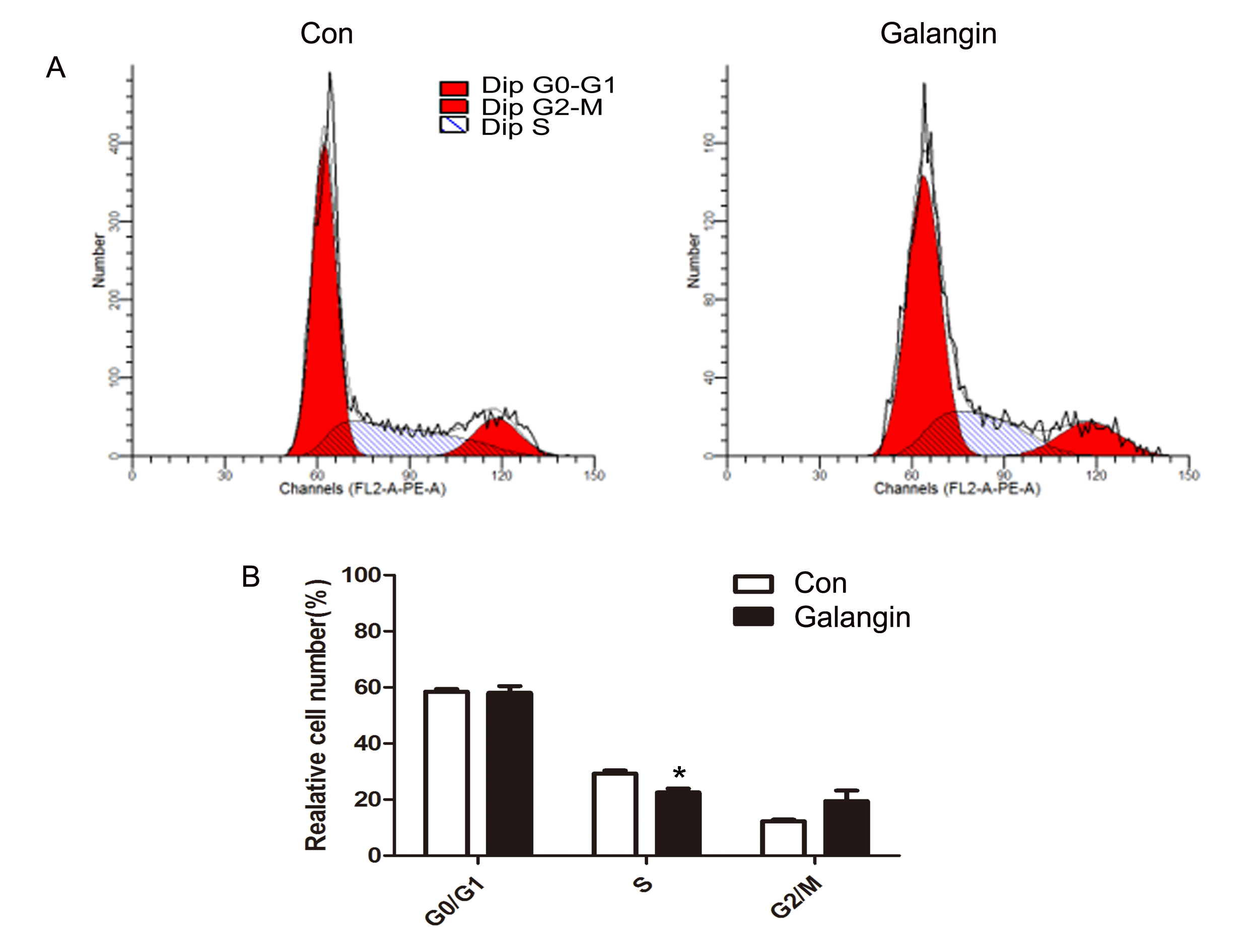

Supplement: Supplementary file 2 — Fig S2 [file CAM4-9-5546-s002.tif]

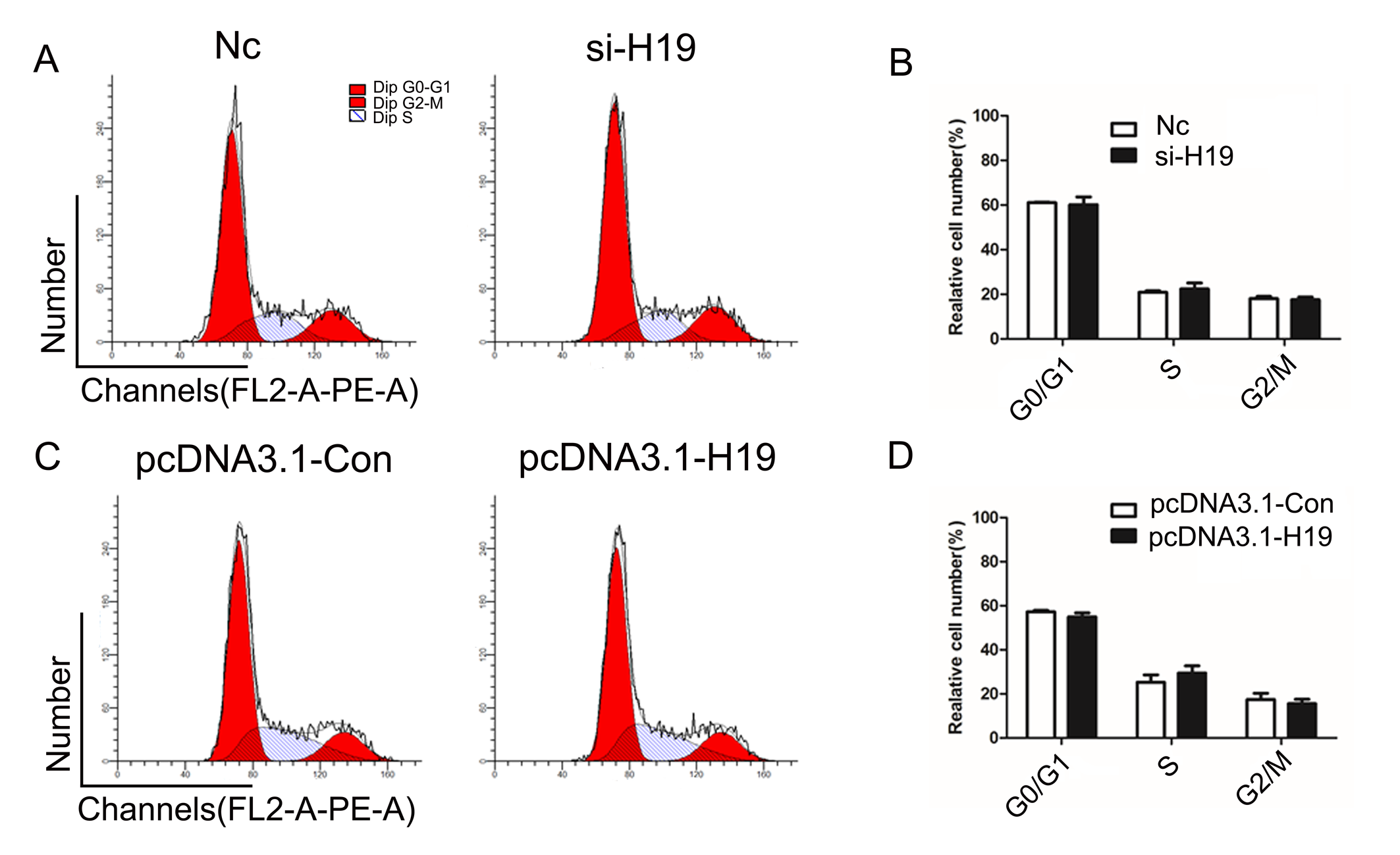

Supplement: Supplementary file 3 — Fig S3 [file CAM4-9-5546-s003.tif]
