# Supplementary material for: Galangin promotes cell apoptosis through suppression of H19 expression in hepatocellular carcinoma cells
Source: Cancer Med. 2020 Jun 2;9(15):5546–57. doi: 10.1002/cam4.3195 (PMC7402821; doi:10.1002/cam4.3195)
Supplement: Supplementary file 4 — Table S1‐S4 [file CAM4-9-5546-s004.docx]

**Table**

Table S1 Sequence of sgRNA

| *SgRNA*s |  | | Sequences (5’→3’) | PAM | | |
| --- | --- | --- | --- | --- | --- | --- |
| SgRNA1 | |  | F: CACCGCTCCTTGCTGCGCAATGTCC  R: AAACGGACATTGCGCAGCAAGGAGC | | CGG |  |
| SgRNA2 | |  | F: CACCGAGCGTCACCAAGTCCACTGT  R: AAACACAGTGGACTTGGTGACGCTC | | GGG |  |
| H19  Identification | |  | F: GGAAGACAGGCAGTGCTC  R: ATGCTGTACTGTCTGCCAAG | |  |  |

Table S2 Primers for qPCR analysis

| Genes | Annealing (°C) | | Primer sequences (5’→3’) |
| --- | --- | --- | --- |
| *PFKL* | | 60 | F: TGGCCTCGTGTTGTGTT  R: GATGGAAGCTCAGCCATGA |
| *TPD52* | | 60 | F: GCCCTGTTCATGGAATCAAATG  R: CCTCTTCAACATTATTCTCAATCGG |
| *ERGIC3* | | 60 | F: AGCCTTGGTGCGTCTTT  R: GGTGCCAAGTCTTTACAGTTTAC |
| *H19* | | 60 | F: TGGTGCACTTTACAACCACTG  R: ATGGTGTCTTTGATGTTGGGGC |
| *CFL1* | | 60 | F: GAGCTCATTCTGGCACTTCT  R: CCTCCAAACCCACACATCT |
| *IARS* | | 60 | F: GTCGTGATATCTTTGTTTCAAGACC  R: ACCTAAGGTGACCTGCCT |
| *CARS* | | 60 | F: CAGTCTTGGGAAGCAACCT  R: GCTCGAGCTCTGTGAGAATC |
| *GAPDH* | | 60 | F: TGGTATCGTGGAAGGACTCA  R: GGGCCATCGACAGTCTTC |

Table S3 Primers for BSP analysis

| Genes | | Annealing (°C) | | Primer sequences (5’→3’) |
| --- | --- | --- | --- | --- |
| *H19* DMR  Outer  *Inner* | 58  58 | | F：TTTTTGGTAGGTATAGAGTT  R：AAACCATAACACTAAAACCC  F: TGTATAGTATATGGGTATTTTTGGAGGTTT  R: TCCCATAAATATCCTATTCCCAAATAACC | |

Table S4 Sequence of miR675 and *H19* exon1

| Gene | sequence |
| --- | --- |
| miR-675-5p  miR-675-3p  *H19* exon1 | TGGTGCGGAGAGGGCCCACAGTG  CTGTATGCCCTCACCGCTCA  ATGGGCCCGTTCCAGGCAGAAAGAGCAAGAGGGCAGGGAGGGAGCACAGGGGTGGCCAGCGTAGGGTCCAGCACGTGGGGTGGTACCCCAGGCCTGGGTCAGACAGGGACATGGCAGGGGACACAGGACAGAGGGGTCCCCAGCTGCCACCTCACCCACCGCAATTCATTTAGTAGCAGGCACAGGGGCAGCTCCGGCACGGCTTTCTCAGGCCTATGCCGGAGCCTCGAGGGCTGGAGAGCGGGAAGACAGGCAGTGCTCGGGGAGTTGCAGCAGGACGTCACCAGGAGGGCGAAGCGGCCACGGGAGGGGGGCCCCGGGACATTGCGCAGCAAGGAGGCTGCAGGGGCTCGGCCTGCGGGCGCCGGTCCCACGAGGCACTGCGGCCCAGGGTCTGGTGCGGAGAGGGCCCACAGTGGACTTGGTGACGCTGTATGCCCTCACCGCTCAGCCCCTGGGGCTGGCTTGGCAGACAGTACAGCATCCAGGGGAGTCAAGGGCATGGGGCGAGACCAGACTAGGCGAGGCGGGCGGGGCGGAGTGAATGAGCTCTCAGGAGGGAGGATGGTGCAGGCAGGGGTGAGGAGCGCAGCGGGCGGCGAGCGGGAGGCACTGGCCTCCAGAGCCCGTGGCCAAGGCGGGCCTCGCGGGCGGCGACGGAGCCGGGATCGGTGCCTCAGCGTTCGGGCTGGAGACGAGG |
